# Daphnauranins C–E, Three New Antifeedants from *Daphne aurantiaca* Roots

**DOI:** 10.3390/molecules23102429

**Published:** 2018-09-21

**Authors:** Sheng Zhuo Huang, Qing Yun Ma, Qi Wang, Hao Fu Dai, Yu Qing Liu, Jun Zhou, You Xing Zhao

**Affiliations:** 1 Hainan Key Laboratory for Research and Development of Natural Products from Li Folk Medicine, Ministry of Agriculture, Institute of Tropical Bioscience and Biotechnology, Chinese Academy of Tropical Agricultural Sciences, Haikou 571101, China; huangshengzhuo@itbb.org.cn (S.Z.H.); maqingyun@itbb.org.cn (Q.Y.M.); wangqii112435@126.com (Q.W.); daihaofu@itbb.org.cn (H.F.D.); 2 State Key Laboratory of Phytochemistry and Plant Resources in West China, Kunming Institute of Botany, Chinese Academy of Sciences, Kunming 650201, China; liuyq11457@163.com

**Keywords:** *Daphne aurantiaca*, Thymelaeceae, sesquiterpenoid, antifeedant

## Abstract

Daphnauranins C–E (compounds **1**–**3**), two sesquiterpenoids and one monoterpenoid were isolated from the roots of *Daphne aurantiaca* Diels. Daphnauranin C is a 9-*O*-13 etherified and hydroperoxy-substituted guaiane sesquiterpenoid, daphnauranin D is a guaiane sesquiterpenoid ketal, and daphnauranin E is a monoterpenoid lactone. Their structures were elucidated by comprehensive analyses of MS, 1D NMR, and 2D NMR spectroscopic data. In an anti-feeding activities test, daphnauranins C–E showed activity against male fruit fly with anti-feeding indexes (AI) up to 39.1, 39.2, and 27.8% respectively, at 1 mM.

## 1. Introduction

**Figure 1:**
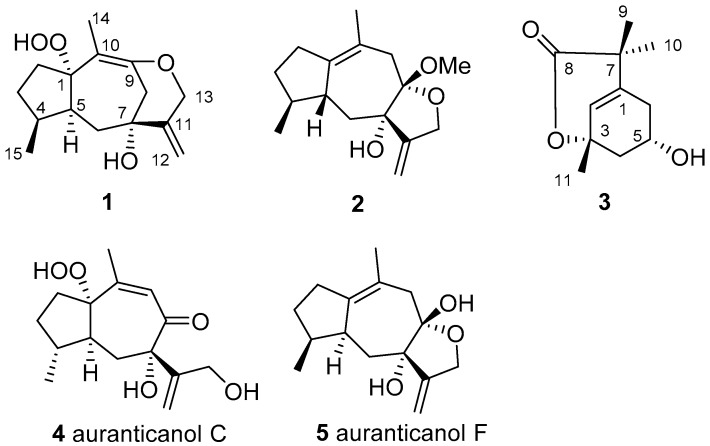
The structures of compounds **1**–**5**.

With the increase of global population and the changes in food-consumption habits, the food production industry is under unprecedented pressure. One of the biggest threats in agriculture are insects, which cause enormous production losses. To solve this problem there is a need for continuous effective prophylaxis and treatment. Therefore, finding and developing new effective and ecologically safe insecticides and insect repellents is one of the most important science topics for chemical and biological researchers in agriculture [1]. With multifarious natural products, traditional herbs have a long history of use as insecticides and insect repellents [2,3]. Natural products have become a treasury to explore for new insecticides or lead compounds with incomparable excellent structural diversity, ecological safety, biodegradability, and sustainability properties [4,5,6,7]. *Daphne aurantiaca* Diels., a traditional herb, was used as the raw material to make insect repellent paper by Tibetan Buddhists [8]. In previous studies, this plant showed some different natural products from other species. A few diterpenoids and several sesquiterpenoids with special skeletons and insect repellent activities were isolated from the stems of this plant [9,10,11,12]. To seek new insecticide and insect repellent from natural products, herein we studied the *D. aurantiaca* roots and found three new compounds, a 9-*O*-13 etherifie and hydroperoxy-substituted guaiane sesquiterpenoid [daphnauranin C (**1**)], a guaiane sesquiterpenoid ketal [daphnauranin D (**2**)], a monoterpenoid lactone [daphnauranin E (**3**)], and two known compounds: auranticanol C (**4**) and auranticanol F (**5**) (Figure 1). The isolation process and structural elucidation details of these new compounds, as well as their anti-insect assay results, are described in this paper.

## 2. Results and Discussion

**Figure 2:**
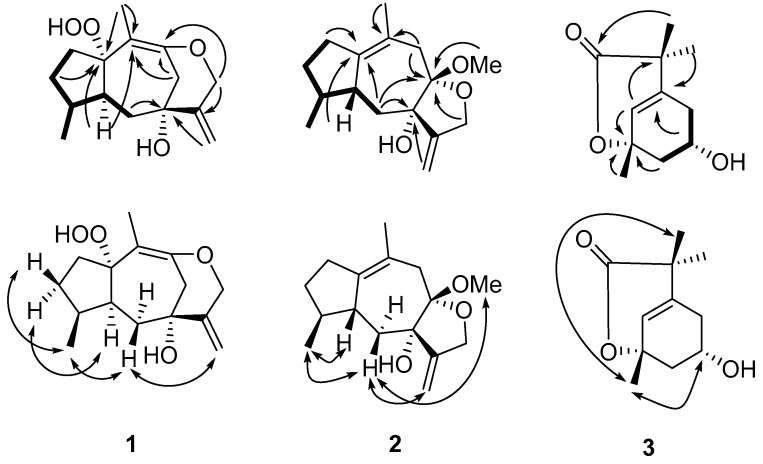
Key ^1^H-^1^H COSY (**–**), HMBC (H→C), and ROESY (↔) correlations of **1**–**3**.

Daphnauranin C (**1**) was isolated as a colorless amorphous powder. Its molecular formula of C_15_H_22_O_4_ was determined by the HRESIMS ion at *m*/*z* 289.1408 [M + Na]^+^ (calcd for C_15_H_22_O_4_Na, 289.1415) (Supplementary Materials), indicative of five degrees of unsaturation. The IR spectrum revealed the presence of hydroxyl (3441 cm^−1^) and double bond (1629 and 1661 cm^−1^) absorptions. The ^1^H-NMR spectrum (Table 1) of compound **1** exhibited signals of two methyls [*δ*_H_ 1.36 (3H, s, H-14) and 0.92 (3H, d, *J* = 6.9 Hz, H-15)], two oxygenated methylene protons (*δ*_H_ 4.54 (1H, d, *J* = 13.2 Hz, Ha-13) and 4.49 (1H, d, *J* = 13.2 Hz, Hb-13), and two olefinic protons (*δ*_H_ 5.32 (1H, d, *J* = 1.3 Hz, Ha-12) and 5.11 (1H, d, *J* = 1.3 Hz, Hb-12). The ^13^C-NMR and DEPT spectroscopic data (Table 1) showed 15 carbon resonances, including two methyls, six methylenes (one olefinic and one oxygenated), two methines, and five quaternary carbons (three olefinic, two oxygenated). According to a comparison of the corresponding NMR data, compound **1** was similar to auranticanol C (**4**) [11], a rare peroxyhydroxyl-substituted guaiane sesquiterpenoid, except for the markedly different shifts at *δ*_C_ 75.8 (s, C-1), 43.4 (t, C-8), 112.4 (s, C-9), and 96.3 (s, C-10) instead of *δ*_C_ 106.7 (s, C-1), 199.1 (s, C-8), 126.1 (s, C-9), and 166.8 (s, C-10) in auranticanol C, indicating compound **1** was generated from auranticanol C by reductive deoxygenation of C-8 and etherification of C-9 to C-13. The determined C_15_H_22_O_4_ molecular formula and the key HMBC correlations from H-14 to C-1 and C-10, from H-8 to quaternary carbon C-10, and from H-8 and H-13 to quaternary carbon C-9 verified this hypothesis. The other ^1^H-^1^HCOSY and HMBC correlations (Figure 2) also confirmed this atom connectivity. The relative configuration of compound **1** was determined to be the same as that of auranticanol C (Figure 2) by ROESY cross-peaks H-3*α* [*δ*_H_ 1.32 (1H, m)] /H-5 [*δ*_H_ 2.46 (1H, m)], H-15/H-3*β* [*δ*_H_ 1.60 (1H, m)], H-15/H-6*β* (*δ*_H_ 1.69 (1H, dd, *J* = 5.2, 13.2 Hz), and H-6*β*/H-12. Thus, the structure of compound **1** was assigned as shown in Figure 1 and it was named daphnauranin C.

Daphnauranin D (**2**) was obtained as a colorless oil, and possessed the molecular formula C_16_H_24_O_3_ based on HRESIMS (*m*/*z* 287.1620 [M + Na]^+^, calcd for C_16_H_24_O_3_Na, 287.1623) with five degrees of unsaturation (Supplementary Materials). The ^13^C-NMR and DEPT spectroscopic data (Table 1) showed 16 carbon resonances classified into two methyls, five methylenes (one olefinic and one oxygenated), two methines, and five quaternary carbons (three olefinic and two oxygenated). Detailed inspection of spectral data of **2** suggested that it was similar to auranticanol F (**5**) (also isolated from stems of *D. aurantiaca*) [11], except for an additional methoxy group (*δ*_C_ 48.7 q) in compound **2**. Compound **2** should thus be generated from auranticanol F (**5**) via C-8 methoxylation as shown, which was further supported by the HMBC correlations (Figure 1) from H-OMe [*δ*_H_ 3.17 (3 H, s)] to C-8 [*δ*_C_ 107.5 (s)] with the aid of its molecular formula C_16_H_24_O_3_. The configuration 7-*α*OH and 8-*β*OMe in **2** was deduced from its ROESY cross-peaks (Figure 2) H-6*β* [*δ*_H_ 1.81 (1H, dd, *J* = 1.3, 14.4 Hz)] to H-8-OMe and H-12 [*δ*_H_ 5.17 (1H, d, *J* = 1.3 Hz)]. The configuration 4-*α*H, 5-*β*H were determined by ROESY cross-peaks (Figure 2) H-15 [*δ*_H_ 0.88 (3H, d, *J* = 7.1 Hz)] to H-6*β* and H-5 [*δ*_H_ 2.18 (1H, m)] with molecular model and comparison of 1D and 2D NMR data to those of auranticanol F. Thus, the structure of compound **2** was assigned as shown and named daphnauranin D.

The molecular formula of daphnauranin E (**3**), was determined as C_11_H_16_O_3_, with four degrees of unsaturation, from HREIMS (*m*/*z* 196.1102 [M]^+^, calcd for C_11_H_16_O_3_, 196.1099 (Supplementary Materials). The ^13^C-NMR and DEPT spectroscopic data (Table 1) showed 11 carbon resonances, including three methyls, two methylenes, two methines (one olefinic and one oxygenated), and four quaternary carbons (one olefinic, one oxygenated, and one carbonyl). By comparing the molecular formula and NMR data (Table 1) with Δ^2^-8-*m*-menthenecarboxylic acid [13], compound **3** was proposed to be derived from this compound via the oxygenation of C-3 and C-5 to a hydroxyl or ester group. The key HMBC correlations (Figure 2) from H-5 [*δ*_H_ 4.31 (1H, dddd, *J* = 2.2, 2.4, 3.2, 3.5 Hz)] to C-1 [*δ*_C_ 178.2 (s)], from H-4 [*δ*_H_ 3.70 (1H, dd, *J* = 2.4, 15.6 Hz) and 3.26 (1H, dd, *J* = 3.2, 15.6 Hz)] to C-3 [*δ*_C_ 86.8 (s)], and from H-2 [*δ*_H_ 5.68 (1H, m)] to C-3, and ^1^H ^1^H COSY correlations between H-4/H-5 [*δ*_H_ 4.31 (1H, dddd, *J* = 2.2, 2.4, 3.2, 3.5 Hz)] and H-5/H-6 [*δ*_H_ 2.48 (1H, dd, *J* = 2.2, 15.4 Hz) and 1.77 (1H, dd, *J* = 3.5, 15.4 Hz)] in compound **3** (Figure 1) supported the assignment. The linkage of 3-*O*-8 lactone in compound **3** was determined by the ^13^C NMR data of C-3 and C-11 [*δ*_C_ 182.6 (s)] and the determined molecular formula C_11_H_16_O_3_. The relative configuration of compound **3** was determined as *β*-Me-11 and *α*-6-OH based on its NOESY NMR spectrum (Figure 2) revealing the key NOE of H-5/H-11 [*δ*_H_ 1.26 (3H, s)]. Thus, the structure of compound daphnauranin E (**3**) was assigned as shown.

Compounds **1**–**3** were all tested for their anti-feeding activities against male fruit fly (*Drosophila melanogaster*) as described before [9,14]. As a result, the anti-feeding index (AI) of **1**–**3** (at the concentration of 1 mM) were 39.1 ± 5.8, 39.2 ± 3.9 and 27.8 ± 6.2%, respectively. Meanwhile AI of the negative control and the positive control (nicotine at 1 mM) were 17.9 ± 2.4 and 28.5 ± 3.9%, respectively (Table 2).

## 3. Experimental

### 3.1. General Information

Optical rotations were acquired on a SEAP-300 polarimeter (Horiba, Kyoto, Japan) ECD were obtained on a Chirascan instrument (Applied Photophysics Ltd., Surrey, UK), UV spectra were measured on a UV 210A spectrophotometer (Hitachi, Hong Kong, China), IR spectra were measured on a FTS-135 spectrometer (Bio-Rad, Hercules, CA, USA) with KBr pellets. 1D and 2D NMR spectra were obtained using an AV400 or DRX-500 instrument (Bruker, Billerica, MA, USA) with TMS as an internal standard, and ESIMS, HRESIMS, and HREIMS were recorded with a Bruker HCT/Esquire (Billerica, MA, USA), VG Auto Spec-3000 mass spectrometer (Manchester, UK) or Autospec Premier spectrometer (Waters, Milford, MA, USA). Column chromatography (CC) was performed on silica gel (200–300 mesh, Qingdao Marine Chemical Inc., Qingdao, China), ODS (40–70 μm, Fuji Silysia Chemical Ltd., Nagoya, Japan), and Sephadex LH-20 (GE Healthcare Bio-Sciences AB, Uppsala, Sweden). Fractions were monitored by TLC and heating after spraying with 7% H_2_SO_4_ in EtOH.

### 3.2. Plant Material

The roots of *Daphne aurantiaca* Diels. were obtained in Shangri-La, Yunnan Province, People′s Republic of China in July 2014,. The voucher specimen (HUANG0008) identified by Prof. Dr. Y. Niu (Kunming Institute of Botany, Chinese Academy of Sciences) was deposited at the Hainan Key Laboratory for Research and Development of Natural Products from Li Folk Medicine, Institute of Tropical Bioscience and Biotechnology, Chinese Academy of Tropical Agriculture Sciences, Haikou, People’s Republic of China.

### 3.3. Extraction and Isolation

The air-dried roots of *D*. *aurantiaca* (2.5 kg) were powdered and extracted with 95% EtOH by refluxing for three hours (3 × 13 L). The combined EtOH solution was concentrated with a rotary evaporator followed by suspension in water (2 L) and successive extraction with EtOAc (3 × 5 L). The EtOAc extract (143 g) was first subjected to silica gel (200–300 mesh, *φ* 16 × 150 cm) CC eluted with CHCl_3_/MeOH (from 50:1 to 1:1, *v*/*v*) to obtain fractions A–C. Fraction B (3.8) was chromatographed repeatedly over a ODS (40–70 μm, *φ* 4 × 18 cm) with MeOH/H_2_O (gradient elution with 20, 30, 40, 50, 60, 70, 80, and 90%, each 500 mL) and Sephadex LH-20 CC (MeOH as solvent) to yield auranticanol F (**5**, 23.2 mg), respectively. Fraction C (43 g) was then subjected to CC over silica gel (200–300 mesh, *φ* 6 × 45 cm) eluted with petroleum ether/acetone (from 3:1 to 0.5:1, *v*/*v*) to give four fractions C1–C4. Fraction C2 (3.8) was chromatographed repeatedly over a ODS (40–70 μm, *φ* 4 × 18 cm) with MeOH/H_2_O (gradient elution with 20, 30, 40, 50, 60, 70, 80, and 90%, each 500 mL) to obtain fractions C2a-C2d. Fractions C2a-C2d was chromatographed repeatedly over Sephadex LH-20 CC, using MeOH as solvent to yield **1** (5.9 mg), **2** (7.9 mg), and **3** (4.3 mg), respectively. Fractions C3 (1.6 g) was chromatographed repeatedly over a ODS (40–70 μm, *φ* 4 × 18 cm) with MeOH/H_2_O (gradient elution with 20, 30, 40, 50, 60, 70, 80, and 90%, each 500 mL) and Sephadex LH-20 CC (MeOH as solvent) to yield auranticanol C (**4**, 5.2 mg).

#### 3.3.1. Daphnauranin C (**1**)

Colorless amorphous powder; [*α*${]}_{D}^{18.2}$+ 36.84 (*c* 0.230, MeOH); UV (MeOH) *λ*_max_ (logε) 202 (3.46); IR (KBr) *ν*_max_ 3441, 2961, 2933, 2872, 1629, 1661, 1439, 1377, 1341, 1305, 1284, 1160, 1048, 1034, 975, 931, 897; ^1^H- and ^13^C-NMR data see Table 1; ESIMS positive *m*/*z* [M + Na]^+^ 289 (80); HRESIMS *m*/*z* [M + Na]^+^ 289.1408 (calcd for C_15_H_22_O_4_Na, 289.1415).

#### 3.3.2. Daphnauranin D (**2**)

Colorless oil; [*α*${]}_{D}^{17.0}$+2.05 (*c* 0.114, MeOH); ECD (MeOH) *∆ε* 273 (+1.15), 356 (−0.094); UV (MeOH) *λ*_max_ (logε) 203 (3.70); IR (KBr) *ν*_max_ 3426, 2955, 2932, 2871, 1712, 1631, 1455, 1434, 1378, 1113, 1035; ^1^H- and ^13^C-NMR data see Table 1; ESIMS positive *m*/*z* [M + Na]^+^ 287 (40); HRESIMS *m*/*z* [M + Na]^+^ 287.1620 (calcd for C_16_H_24_O_3_Na, 287.1623).

#### 3.3.3. Daphnauranin E (**3**)

Colorless amorphous powder; [*α*${]}_{D}^{16.4}$-23.43 (*c* 0.327, MeOH); UV (MeOH) *λ*_max_ (logε) 361 (1.87), 212 (3.57); IR (KBr) *ν*_max_ 3433, 2962, 2931, 2875, 1734, 1627, 1454, 1379, 1264, 1232, 1165, 1051, 1030, 965; ^1^H- and ^13^C-NMR data see Table 1; ESIMS positive *m*/*z* [M + H]^+^ 197 (45); HREIMS *m*/*z* [M]^+^ 196.1102 (calcd for C_11_H_16_O_3_, 196.1099).

### 3.4. Anti-Feeding Activity Bioassay

The anti-feeding activity was tested on male fruit fly (*Drosophila melanogaster* supplied by JoeKai Biotech LLC, Bejing, China) by the feeding counting method as reported in the literature [9,14]. Test compounds, positive control nicotine (98%, Sigma-Aldrich Corporation, St. Louis, MO, USA), and negative control DMSO (dimethylsulfoxide, Sinopharm Chemical Reagent Co., Ltd., Shanghai, China) were dissolved in DMSO to 100 mM, and then diluted to 1 mM with 4% red sugar water. Fifty starved (17 h) male fruit flies were put into one tube for one treatment. Each test was carried out with nine replicates. After 7 min feeding, the number of rubescent abdomen and no-rubescent abdomen fruit fly was counted. The feeding index (FI) was the percentage of rubescent abdomen fruit fly. Thus, the anti-feeding index (AI) was calculated by the following equation AI % = [(1 − FI) × 100].

## 4. Conclusions

Previously, a series of sesquiterpenoids with skeletal diversity revealed the chemical diversity of the *Daphne* genus. The special substituted compounds isolated from *Daphne aurantiaca* also showed the accessibility of biosynthesis. The bioactivity evaluation assay showed that daphnauranins C-E have prominent anti-feeding activities against male fruit fly. Therefore, these three new compounds may be used for potential agricultural chemical development.

## Figures and Tables

**Table 1:** ^1^H (400 MHz) and ^13^C NMR (100 MHz) Data of Compounds **1**–**3** (in CDCl_3_).

	Compound					
	1		2		3	
No.	*δ*_H_ mult. (*J* in Hz)	*δ*_C_	*δ*_H_ mult. (*J* in Hz)	*δ*_C_	*δ*_H_ mult. (*J* in Hz)	*δ*_C_
1	-	75.8s	-	141.0s		178.2s
2	1.68 m 1.63 m	31.7t	1.65 m 1.40 m	32.5t	5.68 s	112.8d
3	1.60 m 1.32 m	31.5t	2.33 m 2.19 m	31.7t		86.8s
4	1.96 m	37.6d	1.88 m	38.4d	1.96 dd (2.4, 15.6) 1.52 dd (3.2, 15.6)	47.2t
5	2.46 m	36.8d	2.18 m	39.7d	4.31 dddd (2.2, 2.4, 3.2, 3.5)	66.7d
6	1.69 dd (5.2, 13.2) 1.54 dd (11.6, 13.2)	32.4t	1.81 dd (1.3, 14.4) 1.66 m	29.2d	2.48 dd (2.2, 15.4) 1.77 dd (3.5, 15.4)	45.6t
7	-	78.9s	-	78.9s		35.9s
8	2.49 d (14.0) 1.86 d (14.0)	43.4t	-	107.5s		182.6s
9	-	112.4s	2.95 d (17.2) 2.26 d (17.2)	33.6t	1.77 s	26.9q
10	-	93.6s	-	120.2s	1.46 s	26.4q
11	-	154.3s	-	152.4s	1.26 s	30.6q
12	5.32 d (1.3) 5.11 d (1.3)	107.6t	5.17 d (1.3) 5.00 d (1.3)	104.6t		
13	4.54 d (13.2) 4.49 d (13.2)	70.0t	4.50 d (13.1) 4.23 d (13.1)	67.1t		
14	1.36 s	25.9q	1.76 s	23.9q		
15	0.92 d (6.9)	14.4q	0.88 d (7.1)	15.6q		
-OMe			3.17 s	48.7q		

**Table 2:** Anti-feeding index (AI) of compounds **1**–**3** against to male fruit fly (*D. melanogaster*).

Compounds	AI (%)
**1**	39.1 ± 5.8
**2**	39.2 ± 3.9
**3**	27.8 ± 6.2
Blank Control	17.9 ± 2.4
Nicotine (Positive Control)	28.5 ± 3.9

## Supplementary Materials

The following are available online at http://www.mdpi.com/1420-3049/23/10/2429/s1.

## References

[1] Hedin P.A., Hollingworth R.M., Masler E.P., Miyamoto J. (1997). Phytochemicals for Pest Control.

[2] Defagó M., Valladares G., Banchio E., Carpinella C., Palacios S. (2006). Insecticide and antifeedant activity of different plant parts of *Melia azedarach* on *Xanthogaleruca luteola*. Fitoterapia.

[3] Hashim M.S., Devi K.S. (2003). Insecticidal action of the polyphenolic rich fractions from the stem bark of *Streblus asper* on *Dysdercus cingulatus*. Fitoterapia.

[4] Miyakado M., Watanabe K., Miyamoto J. (1997). Natural Products as Leads in Structural Modification Studies Yielding New Agrochemicals. Phytochemicals for Pest Control.

[5] Huang S.Z., Kong F.D., Ma Q.Y., Guo Z.K., Zhou L.M., Wang Q., Dai H.F., Zhao Y.X. (2016). Nematicidal Stemona Alkaloids from *Stemona parviflora*. J. Nat. Prod..

[6] Huang S.Z., Zhang X., Ma Q.Y., Peng H., Zheng Y.T., Hu J.M., Dai H.F., Zhou J., Zhao Y.X. (2014). Anti-HIV-1 tigliane diterpenoids from *Excoecaria acertiflia* Didr. Fitoterapia.

[7] Montenegro I., Pino L., Werner E., Madrid A., Espinoza L., Moreno L., Villena J., Cuellar M. (2013). Comparative study on the larvicidal activity of drimane sesquiterpenes and nordrimane compounds against *Drosophila melanogaster* til-til. Molecules.

[8] Kunming Institute of Botany, Chinese Academy of Sciences (1997). Flora of Yunnan.

[9] Huang S.Z., Li X.N., Ma Q.Y., Dai H.F., Li L.C., Cai X.H., Liu Y.Q., Zhou J., Zhao Y.X. (2014). Daphnauranols A–C, new antifeedant sesquiterpenoids with a 5/6/7 ring system from *Daphne aurantiaca*. Tetrahedron Lett..

[10] Huang S.Z., Huang H.N., Ma Q.Y., Mo M.H., Zhu M.L., Dai H.F., Ji Y.P., Wang Q.H., Zhao Y.X. (2015). The Phytochemicals with Antagonistic Activities Toward Pathogens of a Disease Complex Caused by Meloidogyne incognita and Ralstonia solanacearum. J. Pure Appl. Microb..

[11] Huang S.Z., Zhang X., Ma Q.Y., Zheng Y.T., Dai H.F., Wang Q., Zhou J., Zhao Y.X. (2015). Anti-HIV terpenoids from *Daphne aurantiaca* Diels. stems. RSC Adv..

[12] Zhao Y.X., Huang S.Z., Ma Q.Y., Mei W.L., Dai H.F. (2012). Two new daucane sesquiterpenoids from *Daphne aurantiaca*. Molecules.

[13] Wallach O. (1908). Contribution to Our Knowledge of the Terpenes and the Ethereal Oils. (XCII). The Preparation of Ring Hydrocarbons with Semicyclic Linking and their Application to New Syntheses. Justus Liebig’s Ann. Chem..

[14] Huang S.Z., Ma Q.Y., Kong F.D., Guo Z.K., Wang Q., Dai H.F., Liu Y.Q., Zhou J., Zhao Y.X. (2017). Daphnauranins A and B, two new antifeedants Isolated from *Daphne aurantiaca* roots. Fitoterapia.

