# Supplementary material for: Daphnauranins C–E, Three New Antifeedants from Daphne aurantiaca Roots
Source: Molecules. 2018 Sep 21;23(10):2429. doi: 10.3390/molecules23102429 (PMC6222359; doi:10.3390/molecules23102429)
Supplement: Supplementary file 1 [file molecules-23-02429-s001.pdf]

Daphnauranins C – E, Three New Antifeedants from *Daphne aurantiaca* Roots

Sheng Zhuo Huang [1], Qing Yun Ma [1], Qi Wang [1], Hao Fu Dai [1], Yu Qing Liu [2], Jun Zhou *,[2] and You Xing Zhao *,[1]

[1] Hainan Key Laboratory for Research and Development of Natural Products from Li Folk Medicine, Ministry of Agriculture, Institute of Tropical Bioscience and Biotechnology, Chinese Academy of Tropical Agriculture Sciences, Haikou 571101, People's Republic of China; huangshengzhuo@itbb.org.cn (S.Z.H.); maqingyun@itbb.org.cn (Q.Y.M.); xiaonvreoka@qq.com (Q.W.); daihaofu@itbb.org.cn (H.F.D.)

[2] State Key Laboratory of Phytochemistry and Plant Resources in West China, Kunming Institute of Botany, Chinese Academy of Sciences, Kunming 650201, People's Republic of China.; liuyuqing@mail.kib.ac.cn

* Correspondence: junzhou3264@126.com (J.Z.); zhaoyouxing@itbb.org.cn (Y.X.Z.); Tel.: +86-898-66989095; Fax: +86-898-66989095

Molecules 2018, *23*, 2429; doi:10.3390/molecules23102429

Contents

Figure S1. Key ^{1}H-^{1}H COSY (▬), HMBC (H→C), and ROESY (↔) correlations of 1-3
Figure S2 ^{1}H NMR spectrum of compound 1 in $CDCl_3$.
Figure S3 ^{13}C NMR and DEPT spectrum of compound 1 in $CDCl_3$.
Figure S4 HSQC spectrum of compound 1 in $CDCl_3$.
Figure S5 ^{1}H-^{1}H COSY spectrum of compound 1 in $CDCl_3$.
Figure S6 HMBC spectrum of compound 1 in $CDCl_3$.
Figure S7 ROESY spectrum of compound 1 in $CDCl_3$.
Figure S8 (+) ESI-MS spectrum of compound 1 in $CHCl_3$.
Figure S9 ^{1}H NMR spectrum of compound 2 in $CDCl_3$.
Figure S10 ^{13}C NMR and DEPT spectrum of compound 2 in $CDCl_3$.
Figure S11 HSQC spectrum of compound 2 in $CDCl_3$.
Figure S12 ^{1}H-^{1}H COSY spectrum of compound 2 in $CDCl_3$.
Figure S13 HMBC spectrum of compound 2 in $CDCl_3$.
Figure S14 ROESY spectrum of compound 2 in $CDCl_3$.
Figure S15 (+) ESI-MS spectrum of compound 2 in $CHCl_3$.
Figure S16 ^{1}H NMR spectrum of compound 3 in $CDCl_3$.
Figure S17 ^{13}C NMR and DEPT spectrum of compound 3 in $CDCl_3$.
Figure S18 HSQC spectrum of compound 3 in $CDCl_3$.
Figure S19 ^{1}H-^{1}H COSY spectrum of compound 3 in $CDCl_3$.
Figure S20 HMBC spectrum of compound 3 in $CDCl_3$.
Figure S21 ROESY spectrum of compound 3 in $CDCl_3$.
Figure S22 (+) ESI-MS spectrum of compound 3 in $CHCl_3$.

Molecules 2018, 23, 2429; doi:10.3390/molecules23102429

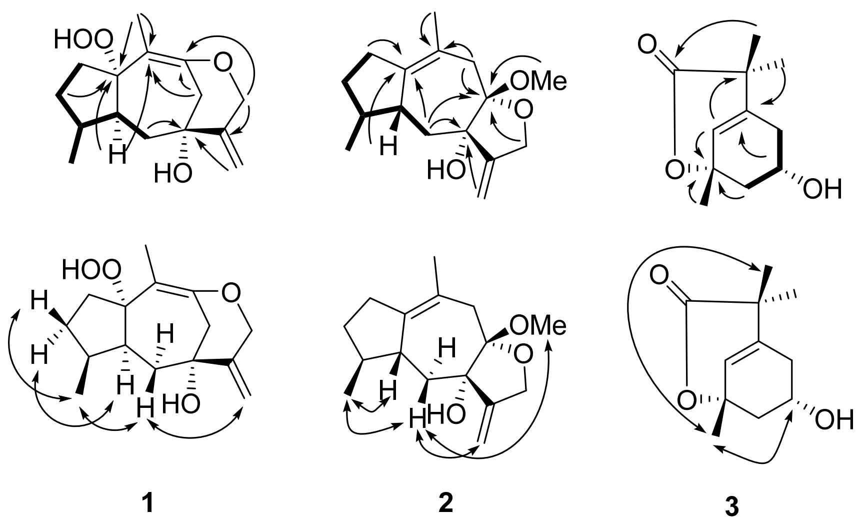

Figure S1. Key ^{1}H-^{1}H COSY (▬), HMBC (H→C), and ROESY (↔) correlations of **1-3**

Molecules 2018, 23, 2429; doi:10.3390/molecules23102429

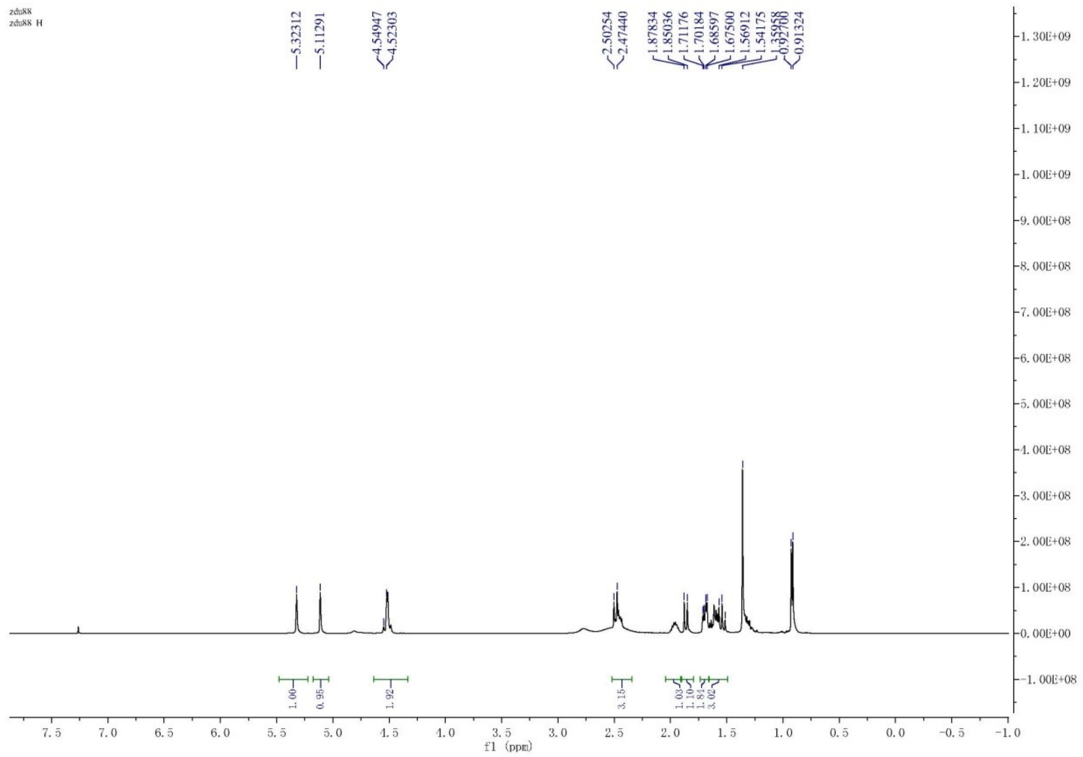

Figure S2 ^{1}H NMR spectrum of compound **1** in $CDCl_3$.

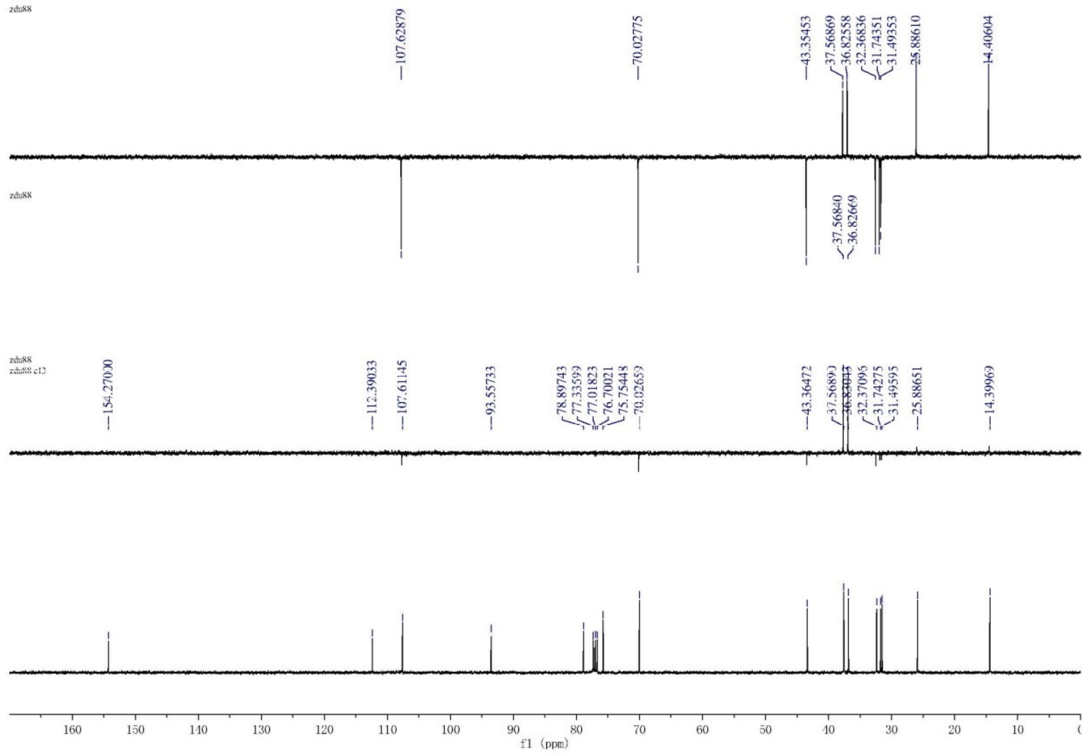

Figure S3 ^{13}C NMR and DEPT spectrum of compound **1** in $CDCl_3$.

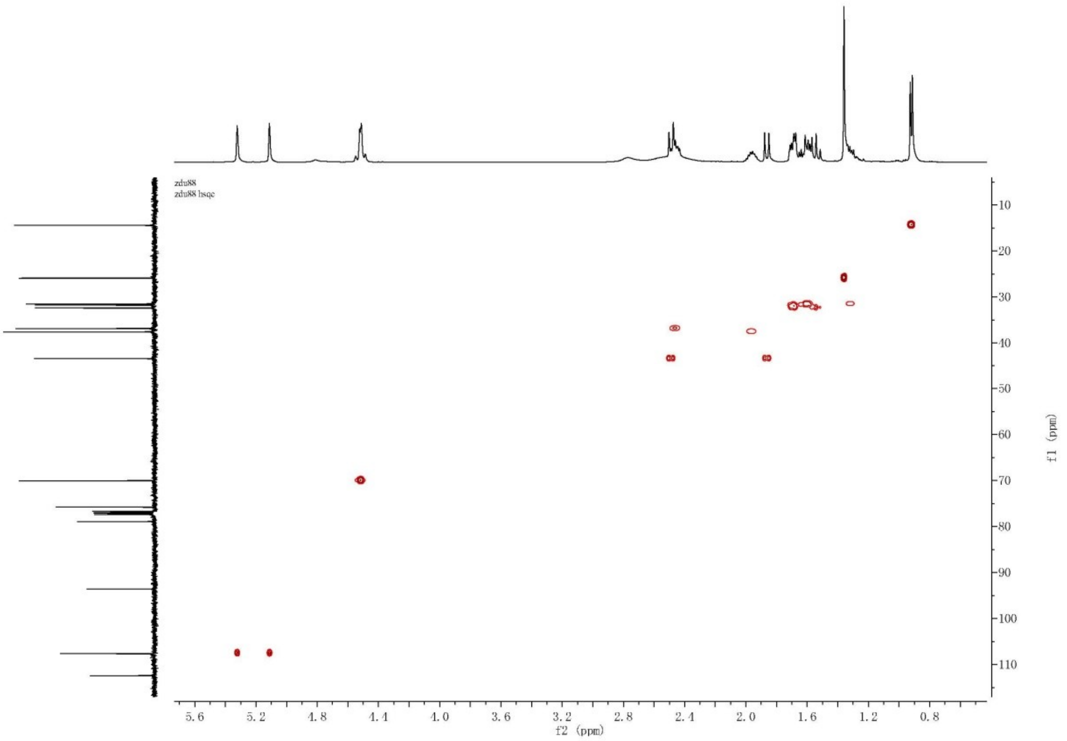

Figure S4 HSQC spectrum of compound **1** in $CDCl_3$.

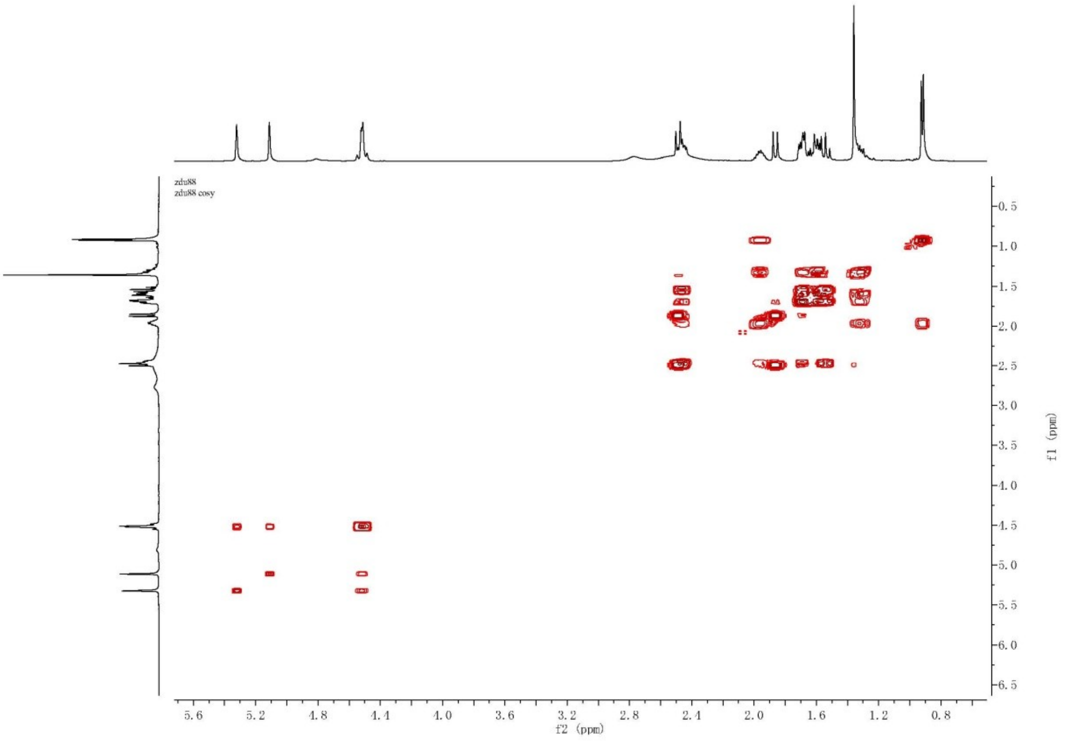

Figure S5 ^1H-^1H COSY spectrum of compound **1** in $CDCl_3$.

Molecules 2018, 23, 2429; doi:10.3390/molecules23102429

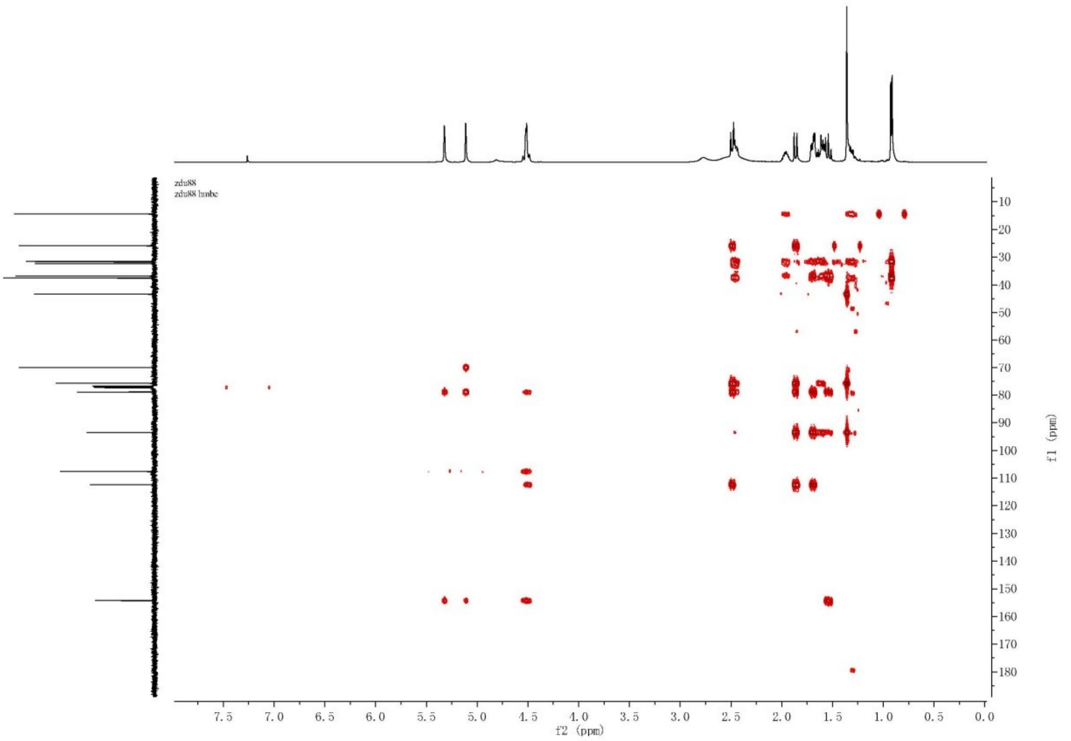

Figure S6 HMBC spectrum of compound **1** in $CDCl_3$.

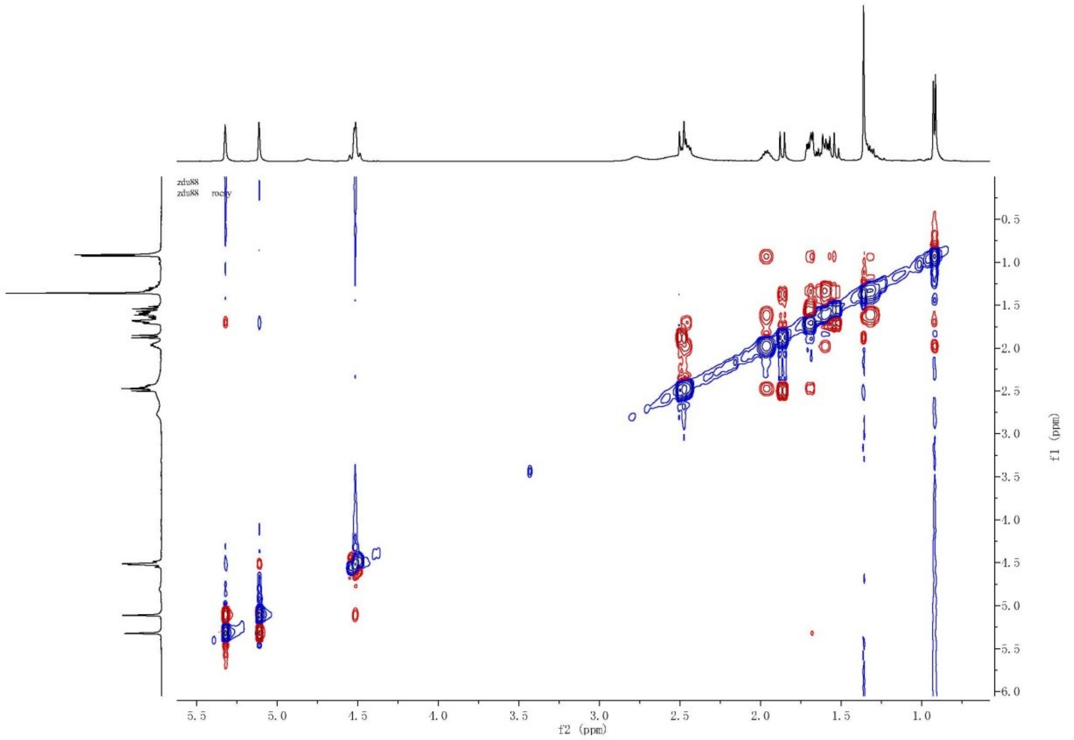

Figure S7 ROESY spectrum of compound **1** in $CDCl_3$.

Molecules 2018, 23, 2429; doi:10.3390/molecules23102429

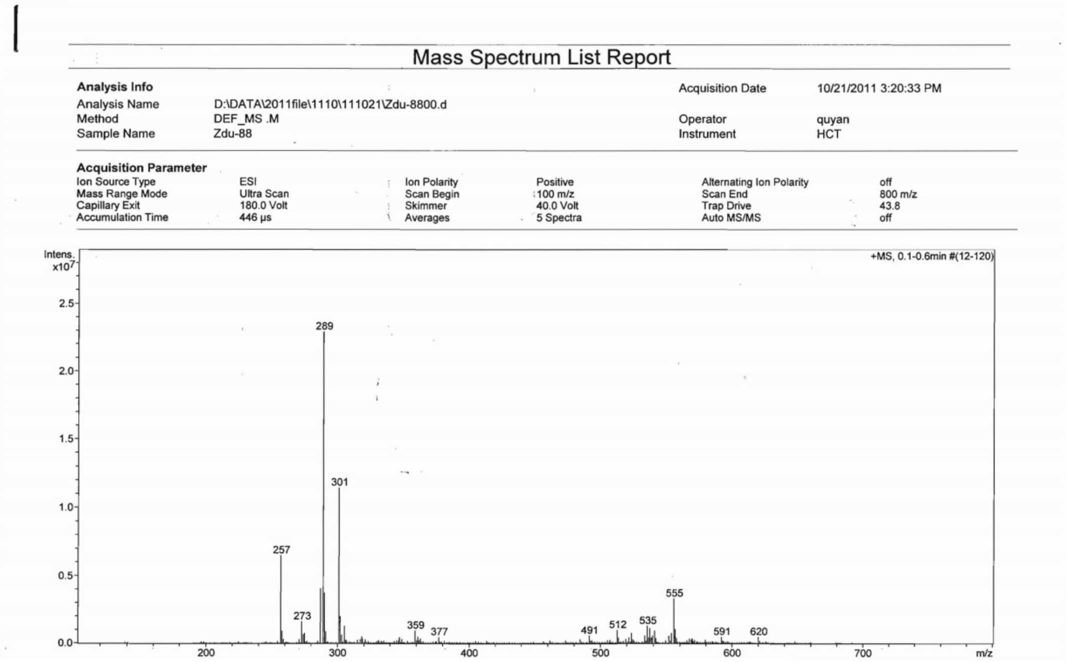

Figure S8 (+) ESI-MS spectrum of compound **1** in $CHCl_3$.

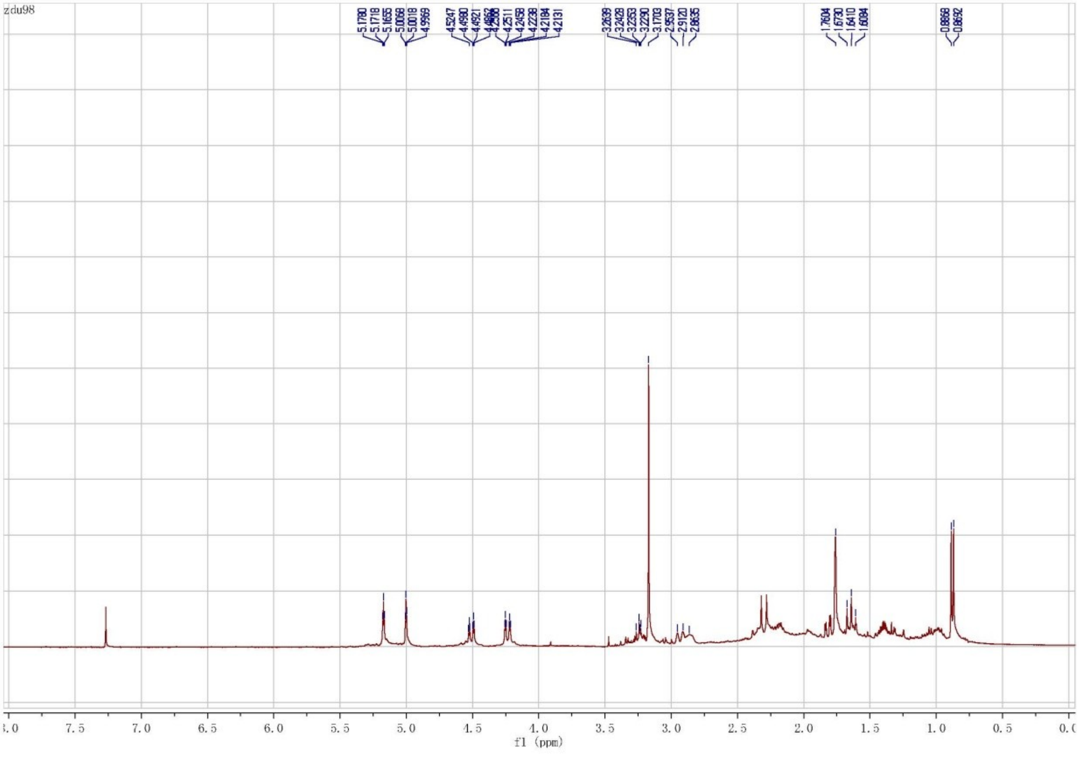

Figure S9 ^{1}H NMR spectrum of compound **2** in $CDCl_3$.

Molecules 2018, 23, 2429; doi:10.3390/molecules23102429

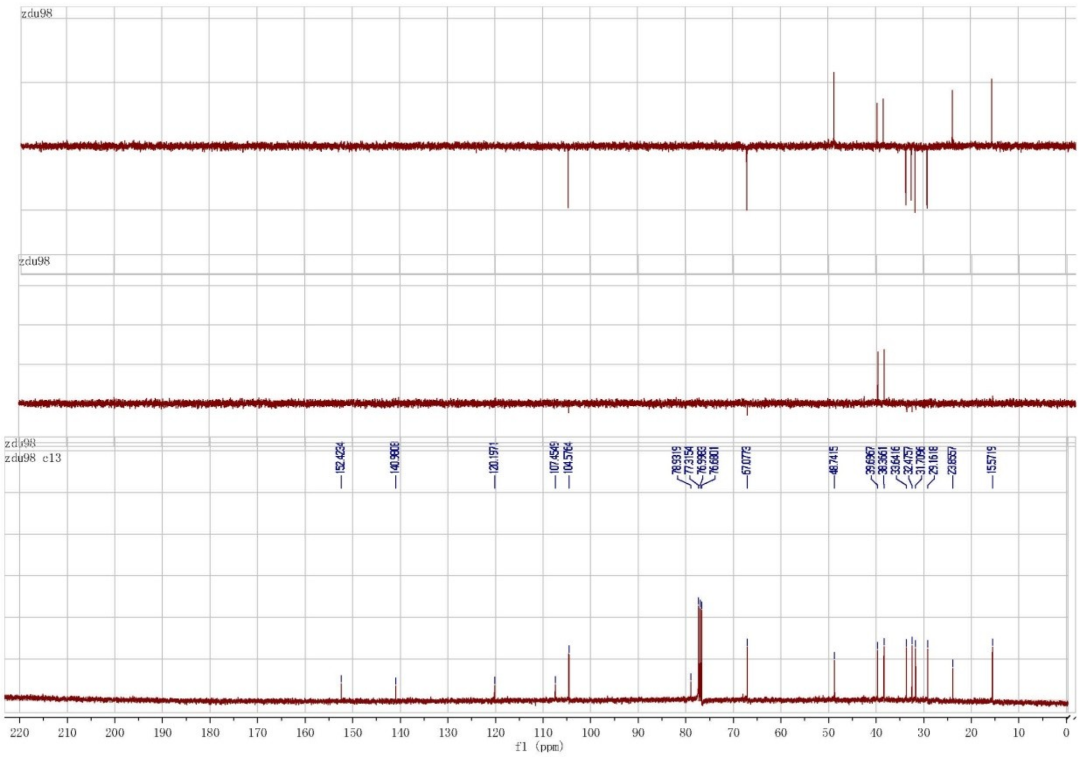

Figure S10 ^{13}C NMR and DEPT spectrum of compound **2** in $CDCl_3$.

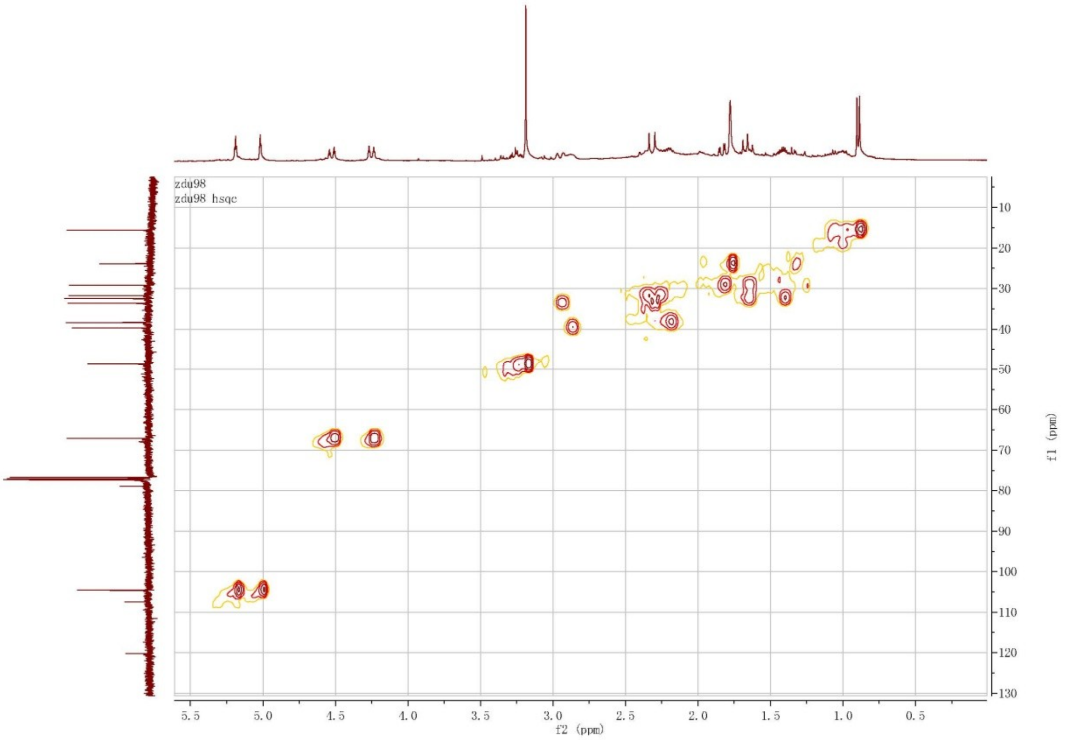

Figure S11 HSQC spectrum of compound **2** in $CDCl_3$.

Molecules 2018, 23, 2429; doi:10.3390/molecules23102429

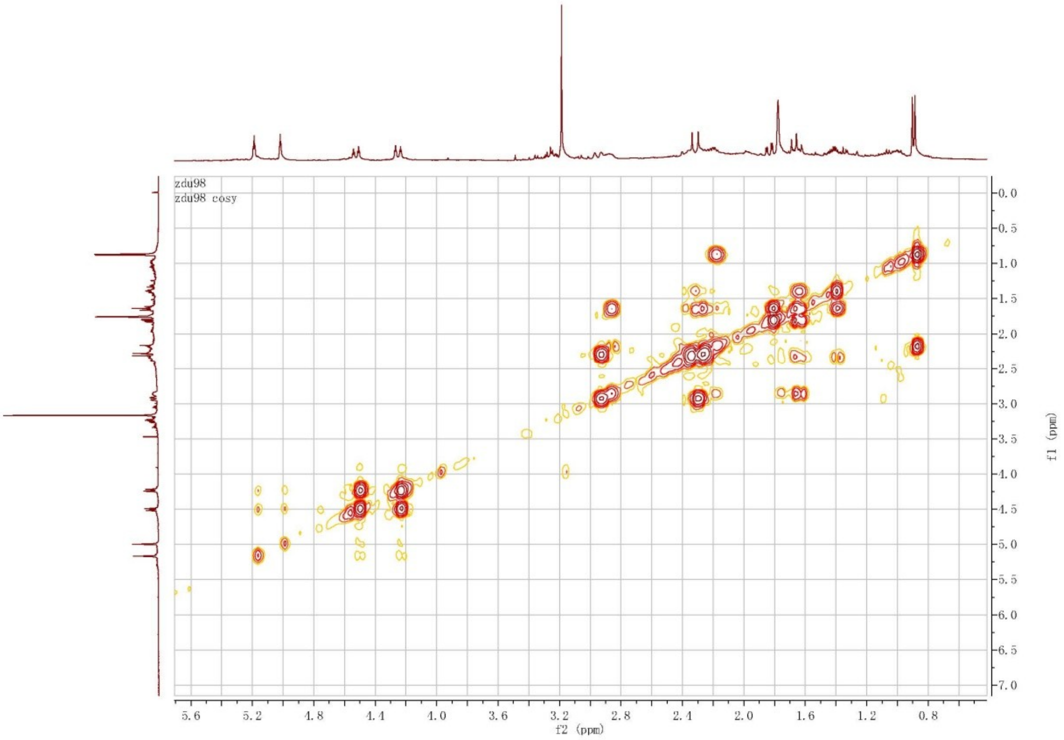

Figure S12 ^{1}H-^{1}H COSY spectrum of compound **2** in $CDCl_3$.

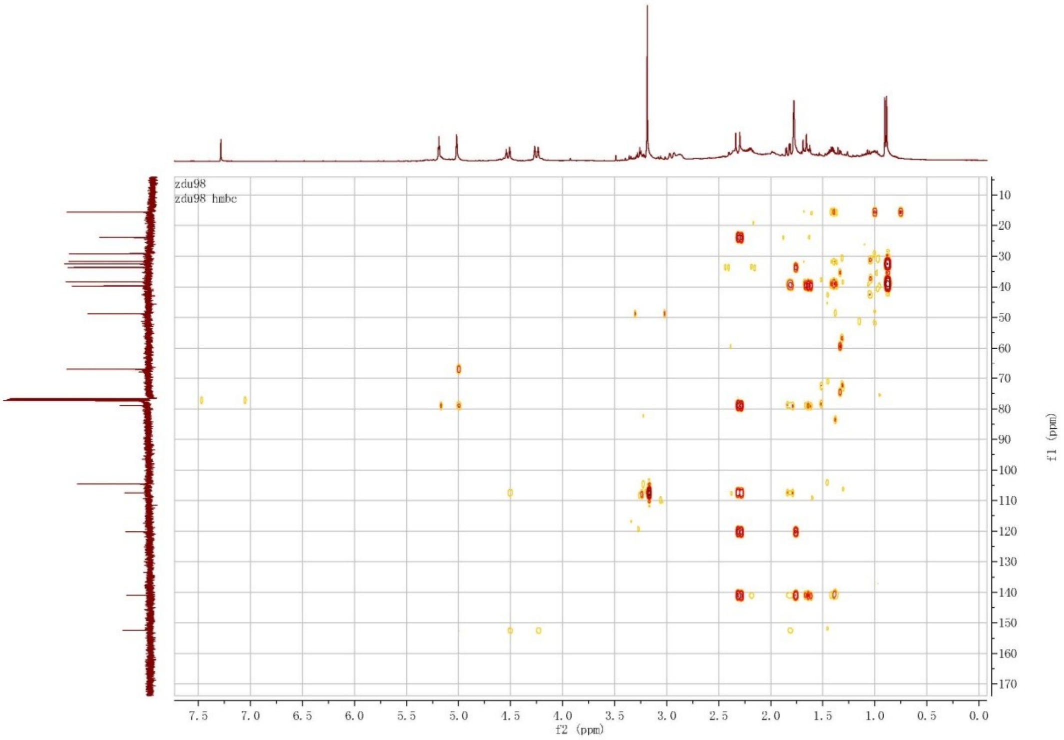

Figure S13 HMBC spectrum of compound **2** in $CDCl_3$.

Molecules 2018, 23, 2429; doi:10.3390/molecules23102429

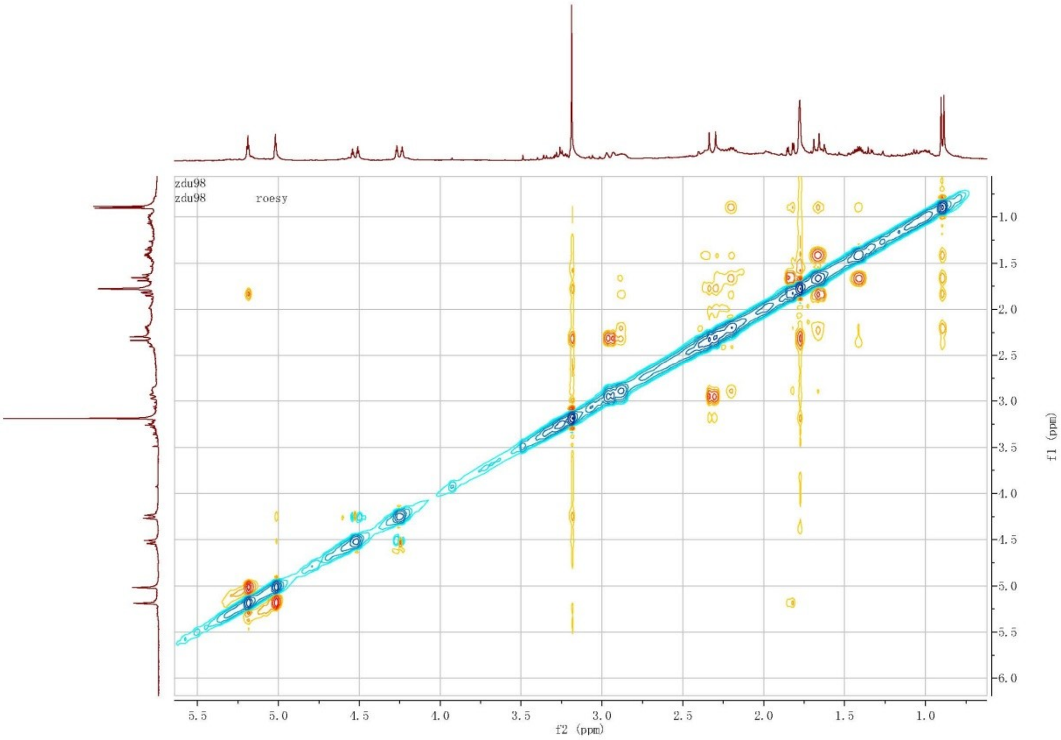

Figure S14 ROESY spectrum of compound **2** in $CDCl_3$.

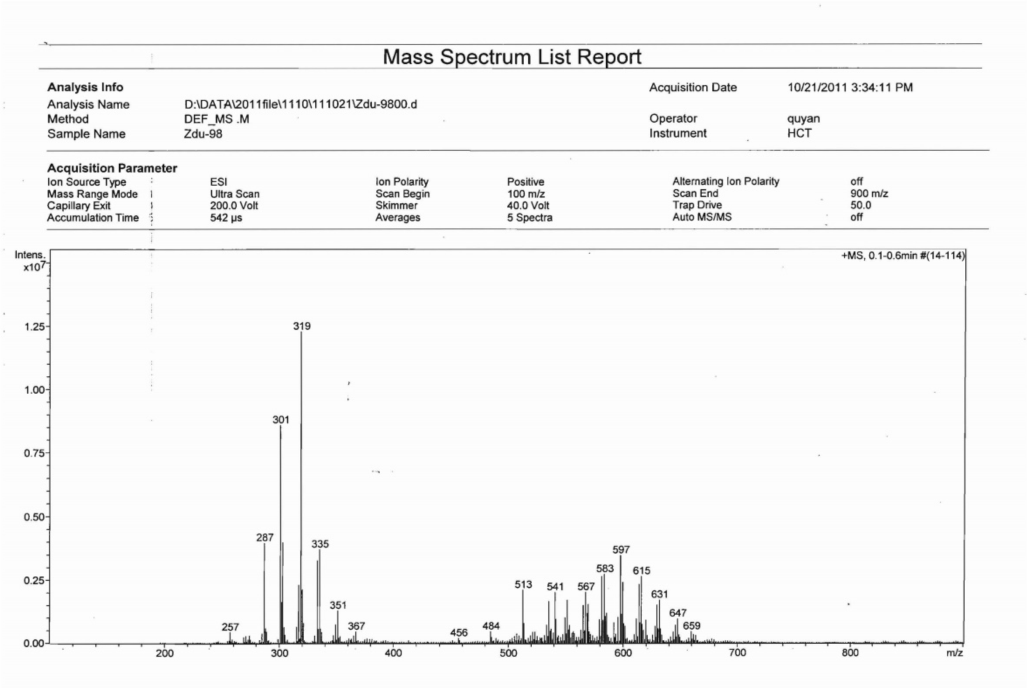

Figure S15 (+) ESI-MS spectrum of compound **2** in $CHCl_3$.

Molecules 2018, 23, 2429; doi:10.3390/molecules23102429

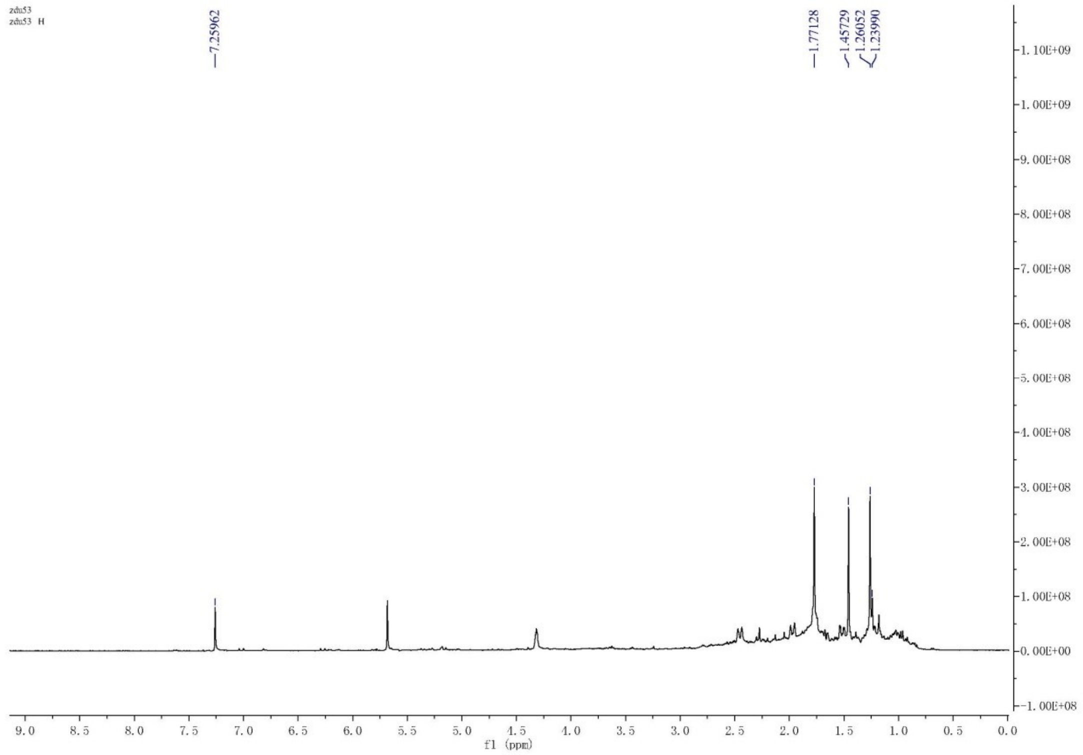

Figure S16 ^{1}H NMR spectrum of compound **3** in $CDCl_3$.

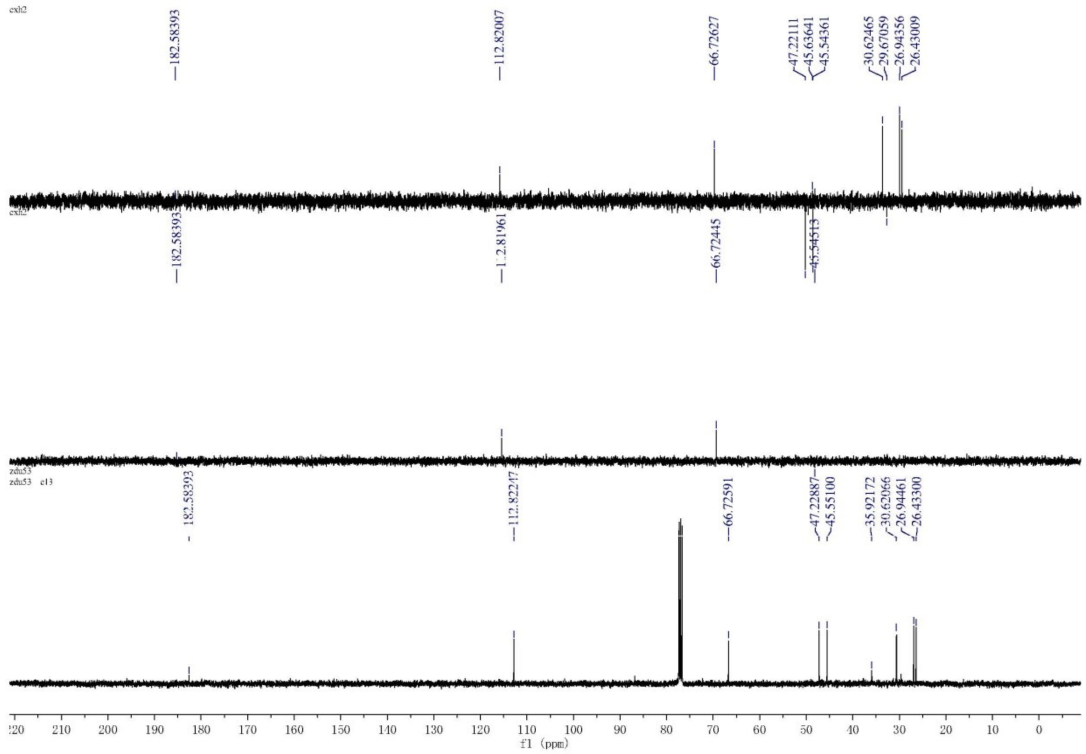

Figure S17 ^{13}C NMR and DEPT spectrum of compound **3** in $CDCl_3$.

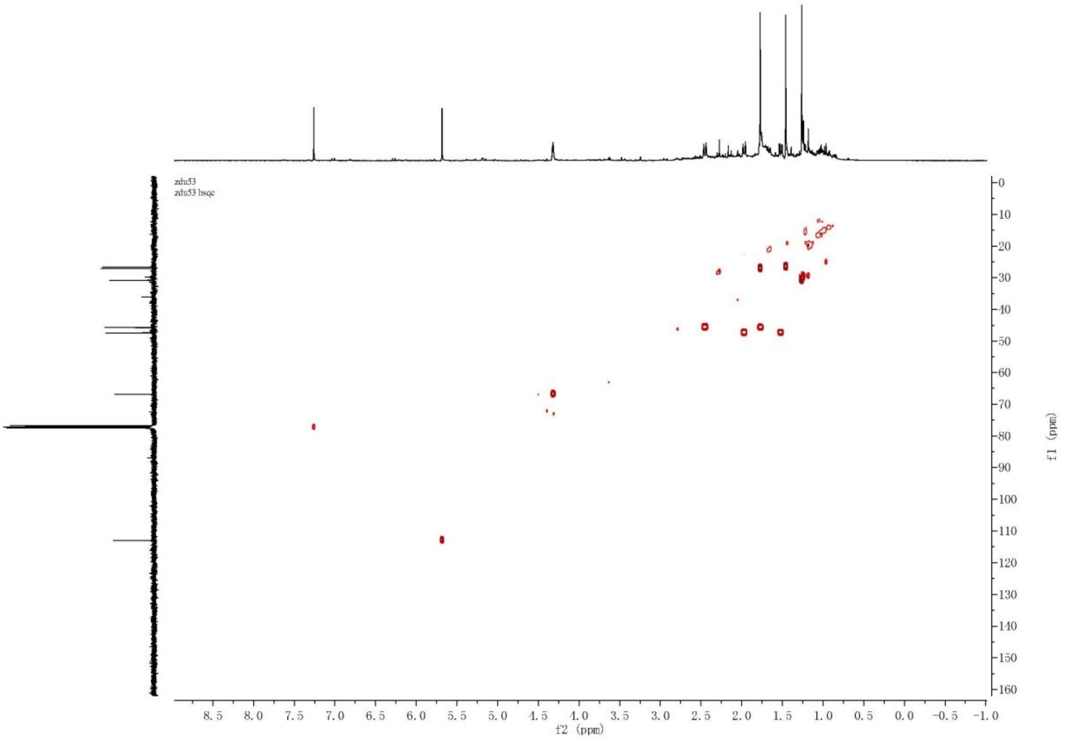

Figure S18 HSQC spectrum of compound **3** in $CDCl_3$.

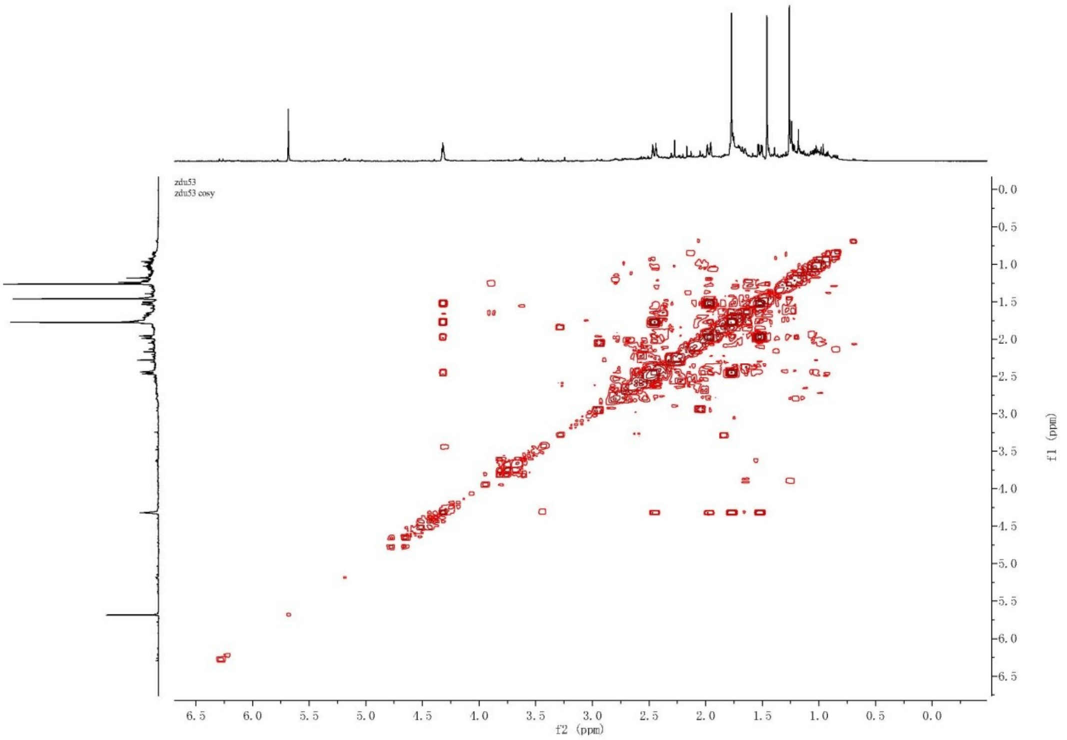

Figure S19 ^1H-^1H COSY spectrum of compound **3** in $CDCl_3$.

Molecules 2018, 23, 2429; doi:10.3390/molecules23102429

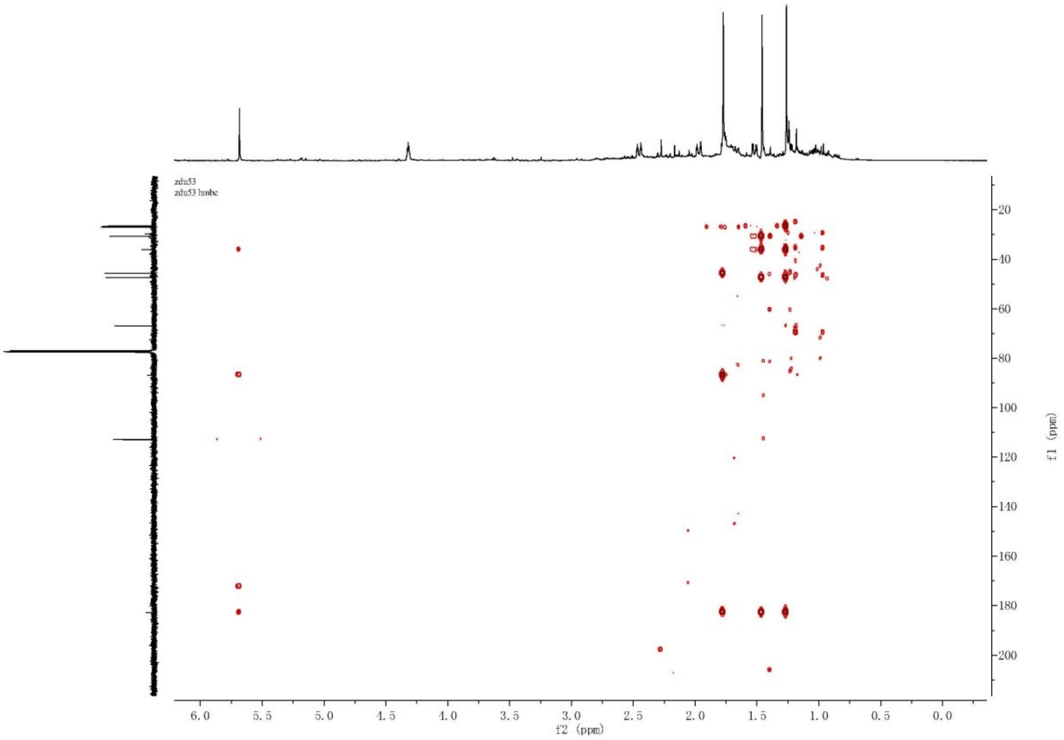

Figure S20 HMBC spectrum of compound **3** in $CDCl_3$.

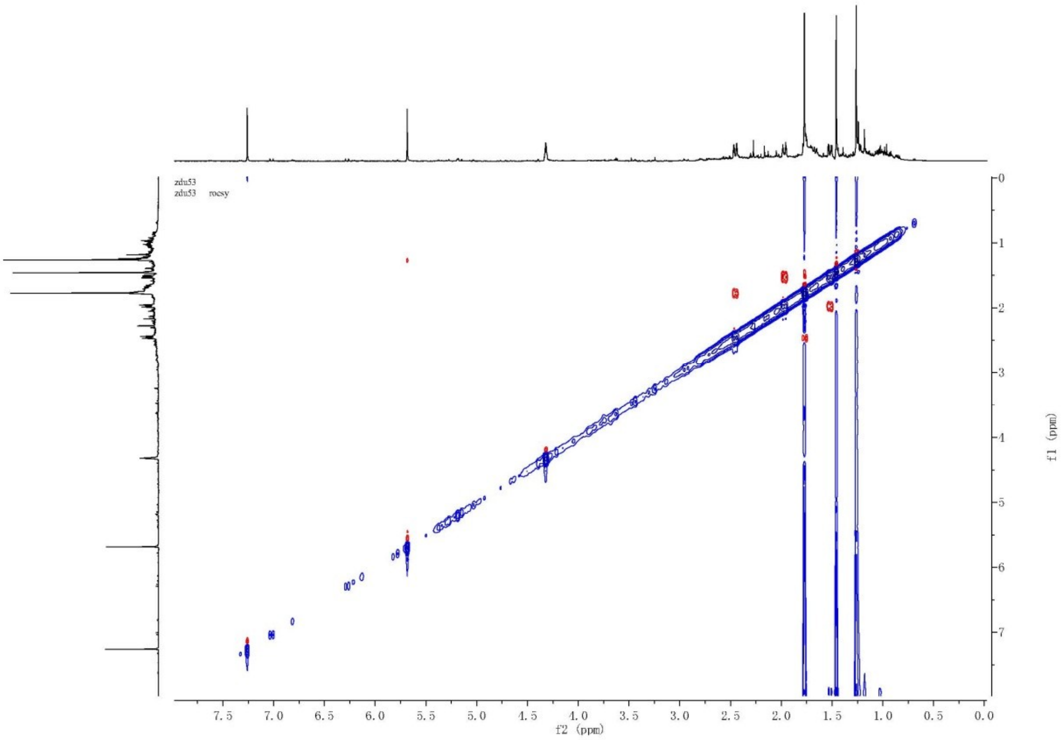

Figure S21 ROESY spectrum of compound **3** in $CDCl_3$.

Molecules 2018, 23, 2429; doi:10.3390/molecules23102429

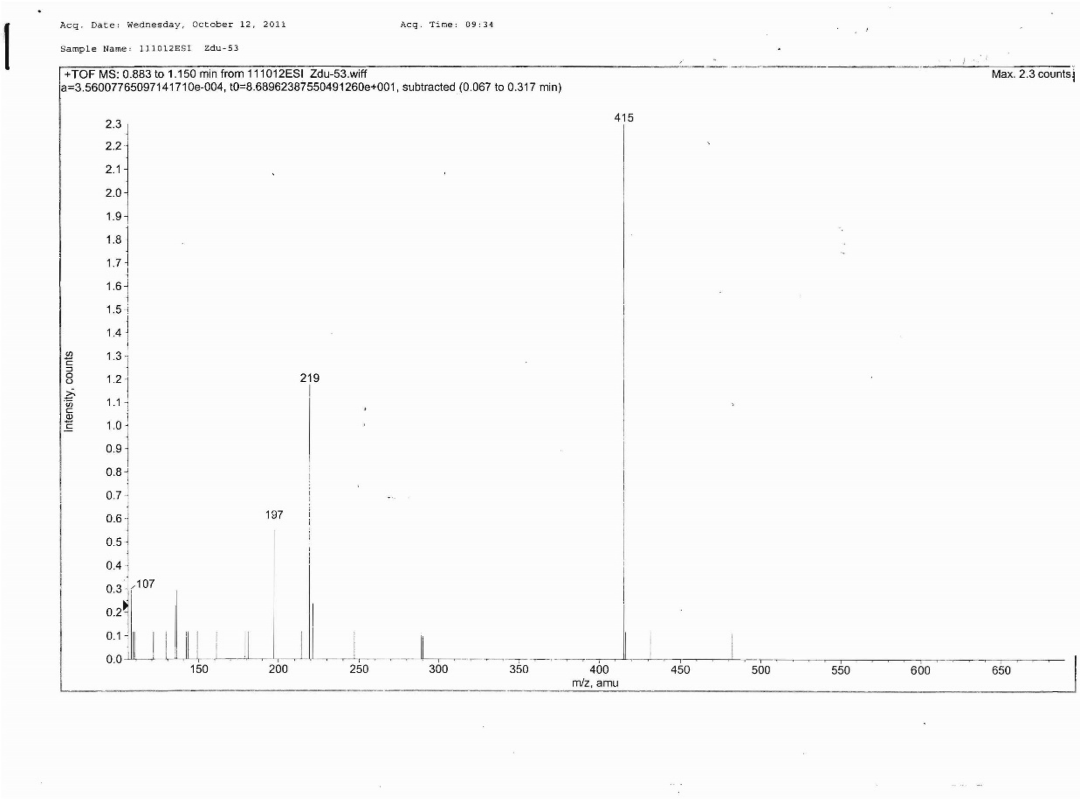

Figure S22 (+) ESI-MS spectrum of compound **3** in $CHCl_3$.

Molecules 2018, 23, 2429; doi:10.3390/molecules23102429
